# Contribution of gut microbiota to metabolism of dietary glycine betaine in mice and in vitro colonic fermentation

**DOI:** 10.1186/s40168-019-0718-2

**Published:** 2019-07-10

**Authors:** Ville M. Koistinen, Olli Kärkkäinen, Klaudyna Borewicz, Iman Zarei, Jenna Jokkala, Valérie Micard, Natalia Rosa-Sibakov, Seppo Auriola, Anna-Marja Aura, Hauke Smidt, Kati Hanhineva

**Affiliations:** 1 Institute of Public Health and Clinical Nutrition, University of Eastern Finland, P.O. Box 1627, FI-70211 Kuopio, Finland; 2 Laboratory of Microbiology, Wageningen University & Research, Stippeneng 4, 6708 WE Wageningen, the Netherlands; 3 JRU Agropolymers Engineering and Emerging Technologies (IATE 1208), SupAgro-INRA-University of Montpellier-CIRAD, Montpellier CEDEX 1, France; 4 School of Pharmacy, University of Eastern Finland, P.O. Box 1627, FI-70211 Kuopio, Finland; 5 VTT Technical Research Centre of Finland, P.O. Box 1000, Tietotie 2, FI-02044 VTT Espoo, Finland

**Keywords:** Betaine, Bran, Colon model, Diet-microbiota interaction, Whole grain

## Abstract

**Background:**

Accumulating evidence is supporting the protective effect of whole grains against several chronic diseases. Simultaneously, our knowledge is increasing on the impact of gut microbiota on our health and on how diet can modify the composition of our bacterial cohabitants. Herein, we studied C57BL/6 J mice fed with diets enriched with rye bran and wheat aleurone, conventional and germ-free C57BL/6NTac mice on a basal diet, and the colonic fermentation of rye bran in an in vitro model of the human gastrointestinal system. We performed 16S rRNA gene sequencing and metabolomics on the study samples to determine the effect of bran-enriched diets on the gut microbial composition and the potential contribution of microbiota to the metabolism of a novel group of betainized compounds.

**Results:**

The bran-enriched study diets elevated the levels of betainized compounds in the colon contents of C57BL/6 J mice. The composition of microbiota changed, and the bran-enriched diets induced an increase in the relative abundance of several bacterial taxa, including *Akkermansia*, *Bifidobacterium*, *Coriobacteriaceae*, *Lactobacillus*, *Parasutterella*, and *Ruminococcus*, many of which are associated with improved health status or the metabolism of plant-based molecules. The levels of betainized compounds in the gut tissues of germ-free mice were significantly lower compared to conventional mice. In the in vitro model of the human gut, the production of betainized compounds was observed throughout the incubation, while the levels of glycine betaine decreased. In cereal samples, only low levels or trace amounts of other betaines than glycine betaine were observed.

**Conclusions:**

Our findings provide evidence that the bacterial taxa increased in relative abundance by the bran-based diet are also involved in the metabolism of glycine betaine into other betainized compounds, adding another potential compound group acting as a mediator of the synergistic metabolic effect of diet and colonic microbiota.

**Electronic supplementary material:**

The online version of this article (10.1186/s40168-019-0718-2) contains supplementary material, which is available to authorized users.

## Background

The past 15 years of research has shown that the modulation of colonic microbial composition by diet [1, 2] is an essential contributor to human health [3–5]. Correspondingly, the microbiota-mediated conversions of a broad array of dietary compounds entering the human body and their impact on the endogenous metabolism thereafter have consequences for health [6–9]. In particular, the role of dietary fibre in shaping the microbiota has become evident, as the low consumption of fibre-rich foods in Western diets has been linked to reduced microbial diversity [10, 11], whereas diets high in dietary plant-based fibre have been associated with increased diversity and the rise of distinct microbial populations [10, 12].

Epidemiological evidence suggests that a diet rich in whole grains reduces the risk of chronic diseases, such as cardiovascular disease, type 2 diabetes, and certain types of cancer [13–16]. In addition, prospective cohort studies have shown that whole grain intake is strongly associated with a linear dose-dependent reduction in the risk of all-cause mortality [17]. Numerous controlled clinical trials have similarly demonstrated the alleviation of chronic disease risk factors [18–22]; however, some studies concluded no improvement in clinical markers, possibly because of differences in the study settings, populations, and defining what is considered as whole-grain food [23–25]. Despite the strong evidence for the beneficial health effects of whole grains, the underlying molecular mechanisms responsible for these effects have remained elusive. Most likely, they are related to the presence of the nutrient-dense bran compartment, rich in fibre, micronutrients, and phytochemicals, retained in whole-grain foods [26–28].

Betaines are zwitterionic compounds with a cationic onium atom, such as quaternary ammonium, that is not bearing any hydrogens. Originally, the term was limited to glycine betaine (*N*,*N*,*N*-trimethylglycine), which was first discovered from sugar beet (*Beta vulgaris* L.) in the nineteenth century, hence giving the name for this compound group. Several betaines are derived from amino acids, and at least 25 such compounds have been detected thus far in living organisms, such as marine algae [29, 30]. The research on the potential health effects of betaines has been mostly concentrated on glycine betaine, as it has an osmoprotective role, protecting the cell from dehydration, osmotic stress, extreme temperatures, and high salinity [31]. In addition, it participates as a methyl donor in transmethylation reactions of the methionine cycle, which in turn occurs in several vital mammalian metabolic processes, such as the transformation of homocysteine into methionine [32]. Trigonelline, the *N*-methylated form of niacin, has been characterized as a plant estrogen [33] and has shown antibacterial (anti-cariogenic) effects in vitro [34]. We have recently conducted a series of investigations and characterized several novel betaines in mice and humans following the consumption of diets rich in whole grains [35, 36]. We also showed that one of the novel betainized compounds, 5-aminovaleric acid betaine (5-AVAB), which accumulated in metabolically active tissues, such as heart and brown adipose tissue (BAT) in mice, can influence mitochondrial energy metabolism in cultured mouse cardiomyocytes by blocking β-oxidation of fatty acids similarly to meldonium, a drug used for ischemia [35, 37]. However, the physiological significance of betaines other than glycine betaine and their relation to maintaining health remains unknown.

Given that the knowledge of betaine metabolism is limited, the aim of this study was to investigate whether colonic microbiota contributes to betaine metabolism by feeding mice with bran-enriched diets, analyzing tissues from conventional and germ-free mice, and performing colonic fermentation of bran in an in vitro model of the human gastrointestinal system. We hypothesized that a (1) diet containing cereal fibre will have an impact on the gut microbial composition and that (2) gut microbiota transforms glycine betaine into other amino acid-derived betaines when the compound reaches the colon within the fibre matrix.

### Results

### Bran-enriched feed causes elevation of betainized compounds in colonic content of mice

We have shown earlier that the inclusion of bran in the diet of mice caused elevation of betainized compounds in urine [36] as well as plasma and tissues [35] of C57BL/6 J mice fed with bran-enriched diets. Herein, we examined colonic contents collected from the same mouse strain fed with similar bran-enriched diets. We used two different parts of whole-grain cereals, rye bran and wheat aleurone (the innermost layer of wheat bran), both of which are abundant in dietary fibre and bioactive compounds [38, 39], such as polyphenols which are known to interact with gut microbiota. Moreover, these two cereal fractions were bioprocessed in order to hydrolyze their dietary fibre and release bioactive compounds, improving their bioavailability in the intestinal tract.

**Fig. 1:**
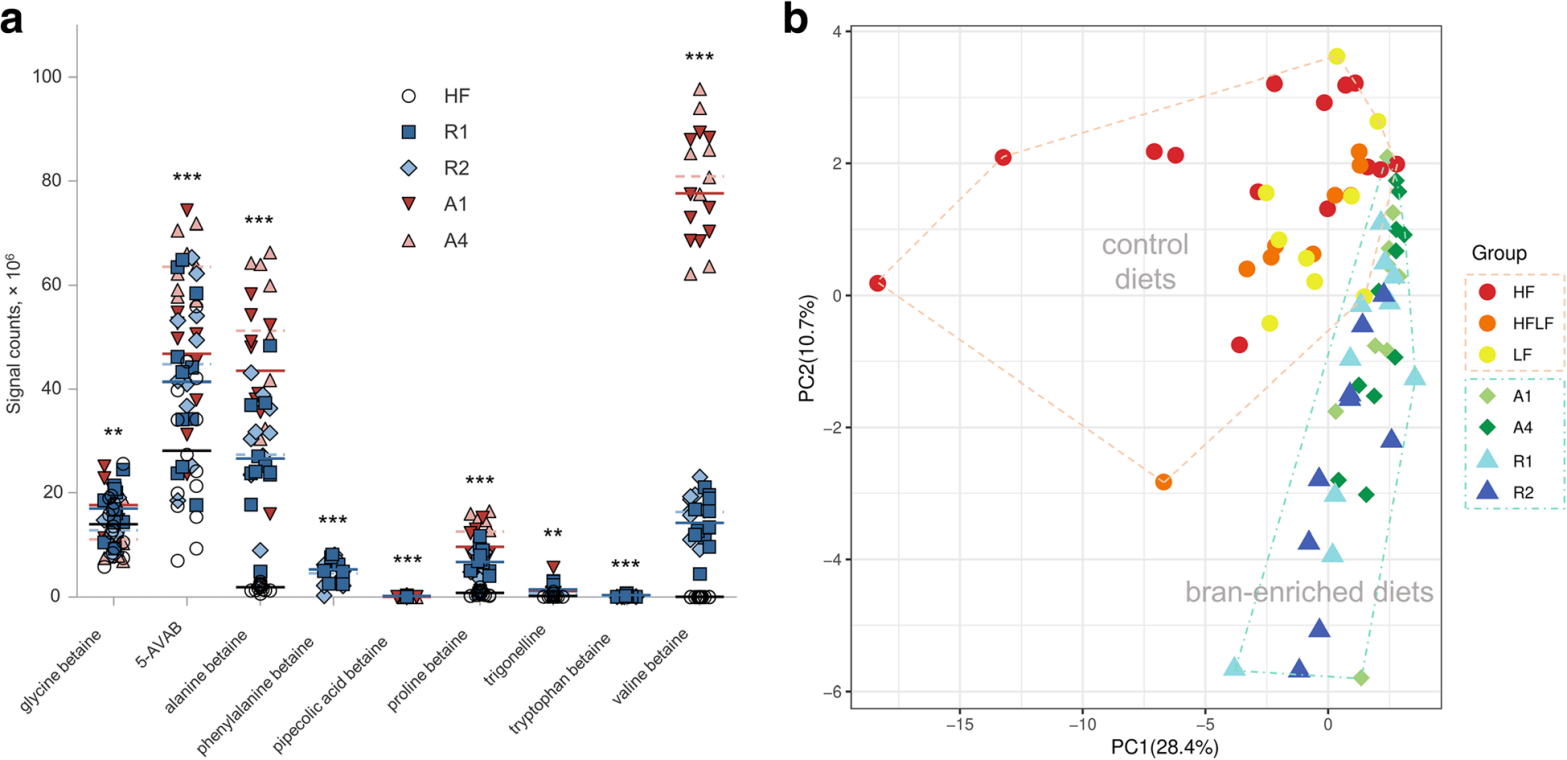
**a** Betaine levels in the colon contents of mice fed with bran-enriched diets. HF high fat (control) diet, R1 native rye bran, R2 bioprocessed rye bran, A1 native wheat aleurone, A4 ground and bioprocessed wheat aleurone. The asterisks signify the *p* value from one-way ANOVA (HF compared to all bran-enriched diets). *p < 0.05; **p < 0.01; ***p < 0.001. **b** Principal component analysis (PCA) of the microbial composition from the caecal contents of mice fed with a high-fat control diet (HF), alternating high-fat low-fat control diet (HFLF), low-fat control diet (LF), wheat aleurone-enriched feed (A1 and A4), and rye bran-enriched feed (R1 and R2)

We found that the concentrations of eight betainized compounds, namely 5-aminovaleric acid betaine (5-AVAB), alanine betaine, phenylalanine betaine, pipecolic acid betaine, proline betaine, trigonelline, tryptophan betaine, and valine betaine, were significantly elevated in the colon content of animals fed with the bran-enriched diets compared to animals fed with the high-fat (HF) control diet (Fig. 1a). Although the majority of the betainized compounds were found also in the samples from mice on the HF diet, pipecolic acid betaine, phenylalanine betaine, and tryptophan betaine were only found in the bran-fed groups, with the latter two compounds being restricted to the rye bran-enriched groups (Fig. 1a). Glycine betaine levels were found to be similar between the animals fed with bran-enriched diets and control diets despite the fact that the bran-enriched diets provide an additional source of glycine betaine.

### Difference in composition of microbiota in mice receiving bran-enriched diets

A qualitative analysis was performed on the microbial composition from the caecal contents of the C57BL/6 J mice, using 16S ribosomal RNA gene sequencing. There were notable differences between the groups receiving the bran-enriched diets and the control groups that included mice on high-fat diet (HF), low-fat diet (LF), or on a high-fat diet switched to low-fat diet after 9 weeks (HFLF) (Fig. 1b). The overall difference in the microbial composition between the bran-enriched diet groups and controls was more evident than the differences between the control groups (Additional file 2: Figure S1). Of the control diets, the mice fed with the LF diet had the closest proximity to mice fed with the bran-enriched diet in the PCA (Fig. 1b). High within-group variability in the bacterial composition was also evident in the analysis. We performed ANOSIM of weighted and unweighted UniFrac distances to further confirm the difference between the control and treatment diets (weighted *R* = 0.231, unweighted *R* = 0.697, *p* < 0.001 for both tests) and between all diets (weighted *R* = 0.321, unweighted *R* = 0.556, *p* < 0.001 for both tests).

**Fig. 2:**
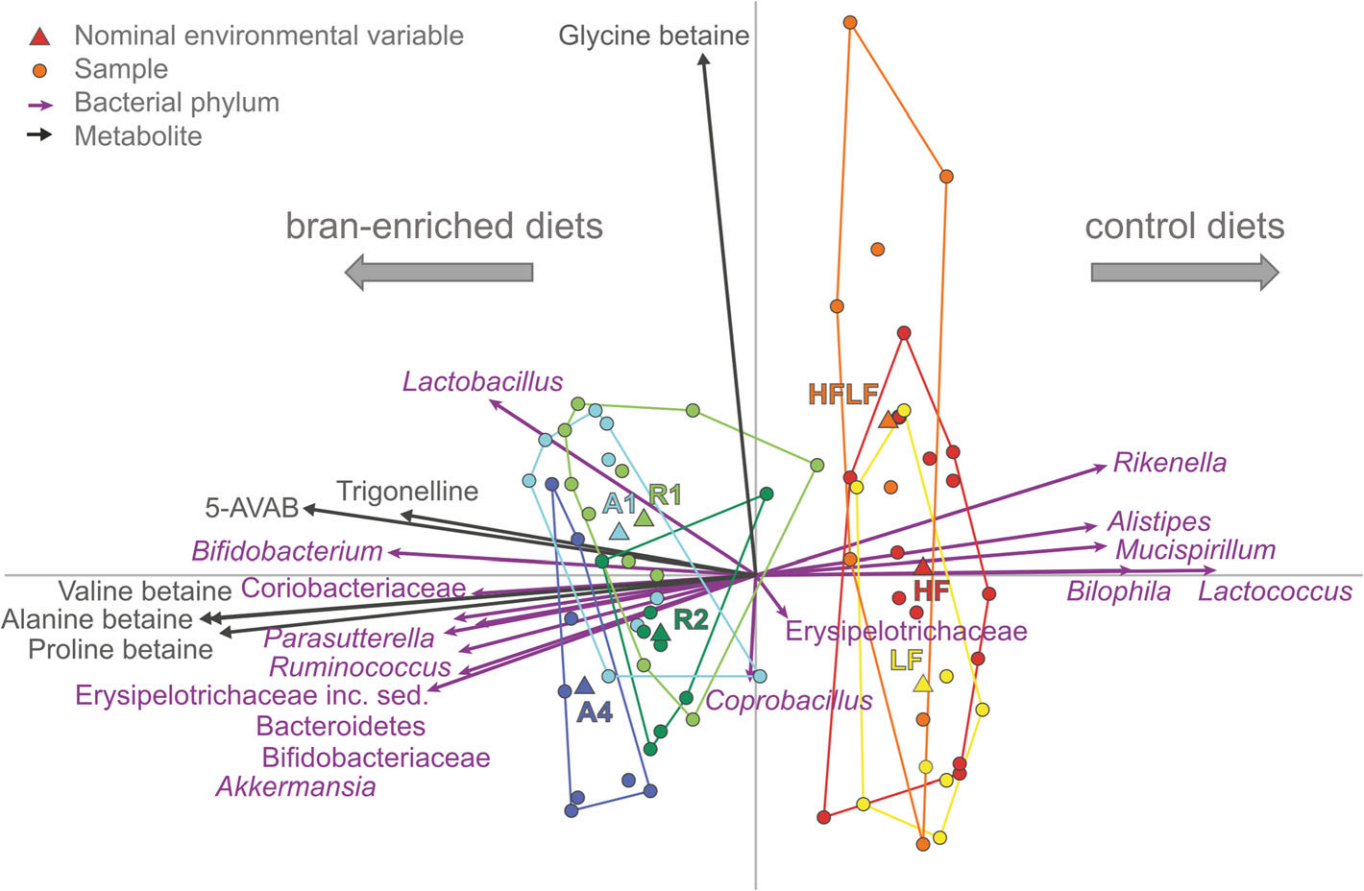
Redundancy analysis (RDA) of the identified betainized compounds (dark grey arrows), diet groups (coloured nodes), and genus-level relative bacterial abundances (purple arrows) in the colonic contents of the studied mice. The microbial composition and the diet groups are used as explanatory variables, together accounting for 93% of the variation in the metabolite levels

**Fig. 3:**
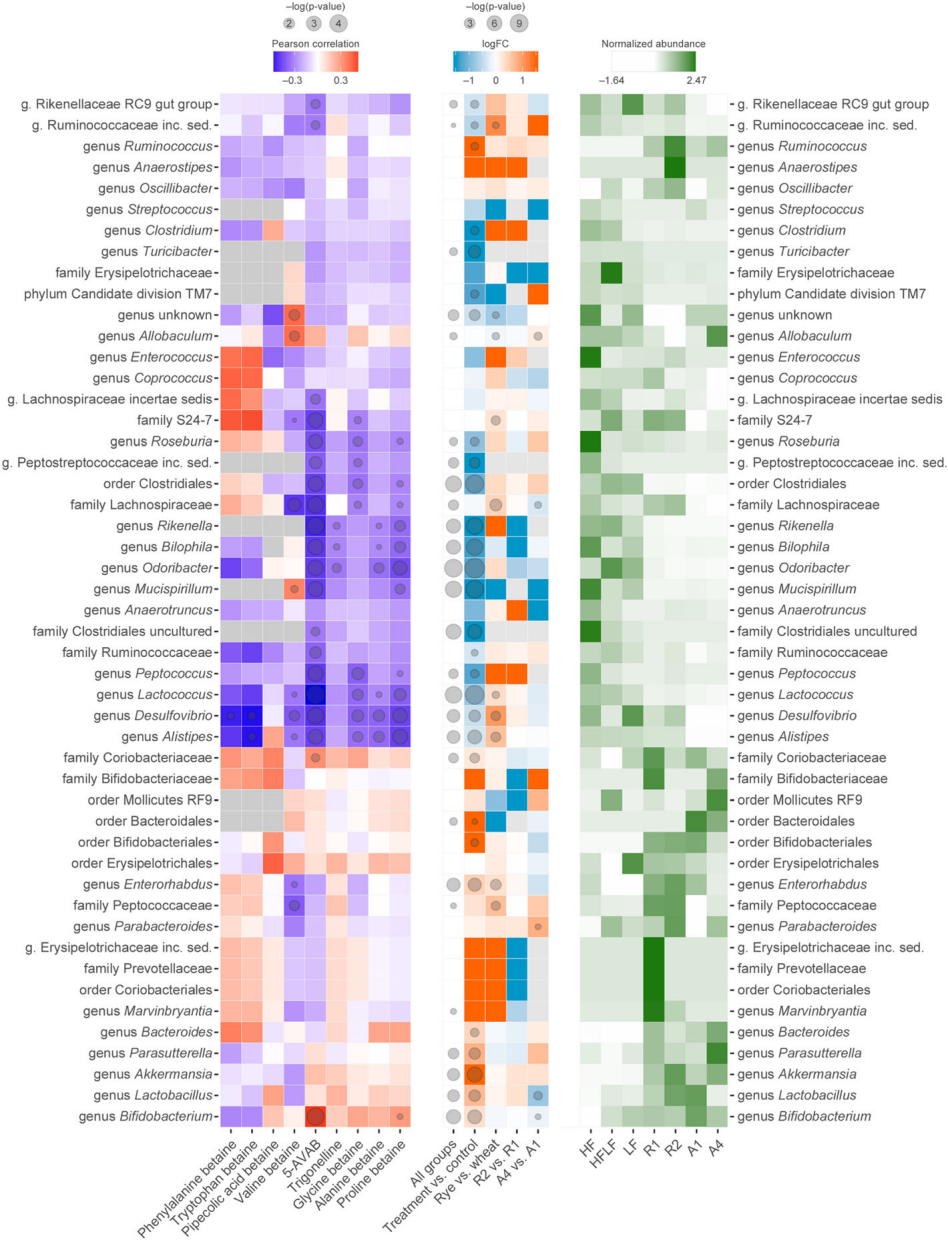
Heat map representation of the identified 48 bacterial taxa and their relation to the identified betaine compounds and the diets. Left: correlations between the relative abundance of microbial taxa in the caecal contents of mice and betaine compounds in the caecal tissue (Pearson correlation, *p* < 0.05 marked with a circle) in HF and bran-enriched diet groups. Centre: comparison of all diet groups (Kruskal–Wallis one-way ANOVA, *p* < 0.05 marked with a circle) and fold changes of the relative abundances of microbial taxa between the bran-enriched treatment diets and the control diets, between the rye bran and wheat aleurone-enriched diets, and between the diets containing unprocessed and bioprocessed rye bran or wheat aleurone (Mann–Whitney *U* test, *p* < 0.05 marked with a circle). Right: normalized average bacterial abundances in each diet group

Redundancy analysis (RDA) and univariate fold change analysis revealed that certain microbial groups, including *Akkermansia*, *Bacteroides*, *Bifidobacterium*, an unclassified genus within family *Coriobacteriaceae*, *Enterorhabdus*, *Lactobacillus*, *Parasutterella*, and *Ruminococcus*, were higher in relative abundance in the groups receiving the bran-enriched diet (Figs. 2 and 3, Additional file 3: Figure S2)*.* In contrast, *Alistipes*, an unclassified genus within the order *Clostridiales*, *Desulfovibrio*, *Mucispirillum*, *Odoribacter*, and *Rikenella* were higher in the control diets. The rye bran-enriched diets caused a higher increase in the relative abundance of several bacterial taxa, including *Alistipes*, *Desulfovibrio*, *Lactococcus*, and unclassified genera within families *Lachnospiraceae* and *Peptococcaceae*, when compared to the wheat aleurone-enriched diets. Bran bioprocessing, which enhances the bioavailability of fibre-bound compounds [40], increased the relative abundance of *Allobaculum* and *Parabacteroides* and decreased the relative abundance of *Bifidobacterium*, an unclassified genus within family *Lachnospiraceae*, and *Lactobacillus* in mice receiving bioprocessed wheat aleurone. In mice receiving either unprocessed or bioprocessed rye bran, there were no statistically significant differences in the relative bacterial abundances. A few taxa were higher in relative abundance only in a certain diet group, such as *Roseburia* in the HF diet group and *Allobaculum* and *Parasutterella* in the group receiving a bioprocessed wheat aleurone-enriched diet (A4) (Fig. 3, Additional file 3: Figure S2). Unclassified genera within families *Prevotellaceae* and *Erysipelotrichaceae* were only found from the caecal contents of one study animal that had received the diet enriched with unprocessed rye bran (R1).

**Fig. 4:**
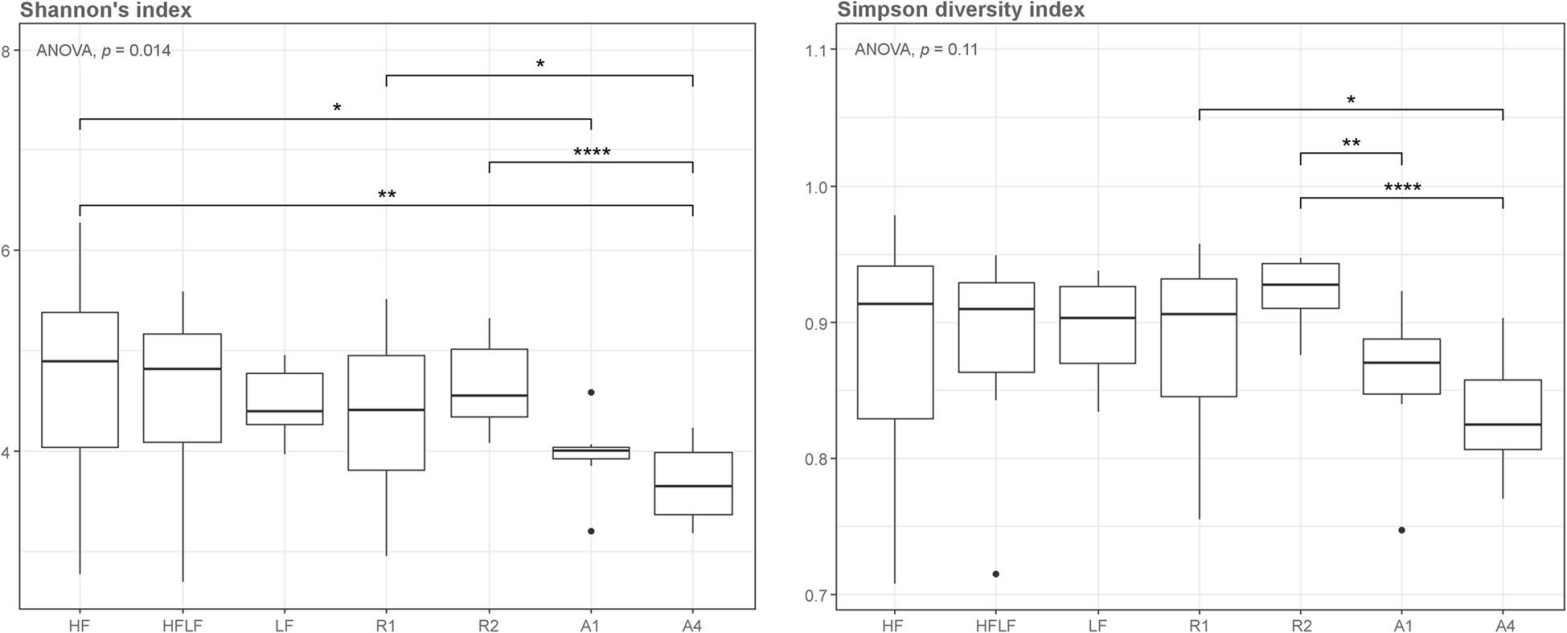
Alpha diversity of the caecal microbiota in the C57BL/6 J mice on the different control and treatment diets, measured according to Shannon’s index (left) and Simpson diversity index (right)

We also measured the alpha diversity of the caecal microbiota in the C57BL/6 J mice to assess the impact of diet on the richness and diversity of the microbial population. The diversity was significantly lower in the wheat aleurone-based A1 and A4 diets compared to the rye-enriched R1 and R2 diets (Shannon’s index and Simpson diversity index) and compared to the HF diet (only for Shannon’s index) (Fig. 4). No significant difference in diversity was observed between the control and rye-enriched diets.

### Correlation between gut microbiota and individual betainized compounds

After finding marked differences in the content of betainized compounds as well as the microbial composition after the consumption of bran-enriched feed, we analyzed whether there were any specific correlations between the microbial taxa and betainized compounds. We observed that the increment of the bacterial taxa associated with the bran-enriched diets was associated with the levels of betainized compounds, including 5-AVAB, proline betaine, and valine betaine, in the caecal tissue of mice (Figs. 2 and 3). However, glycine betaine levels did not clearly show any association with any of the treatment groups and were not positively associated with any bacterial taxa. On the other hand, a strong positive correlation was found between 5-AVAB and *Bifidobacterium*, and strong inverse correlations were observed between 5-AVAB and 18 different bacterial taxa, including *Alistipes*, unclassified genus within the *Clostridiales*, *Desulfovibrio*, *Mucispirillum*, *Odoribacter*, and *Rikenella*, with decreased relative abundance in the bran-enriched diet groups (Fig. 3).

### Levels of betainized compounds in germ-free mice

**Fig. 5:**
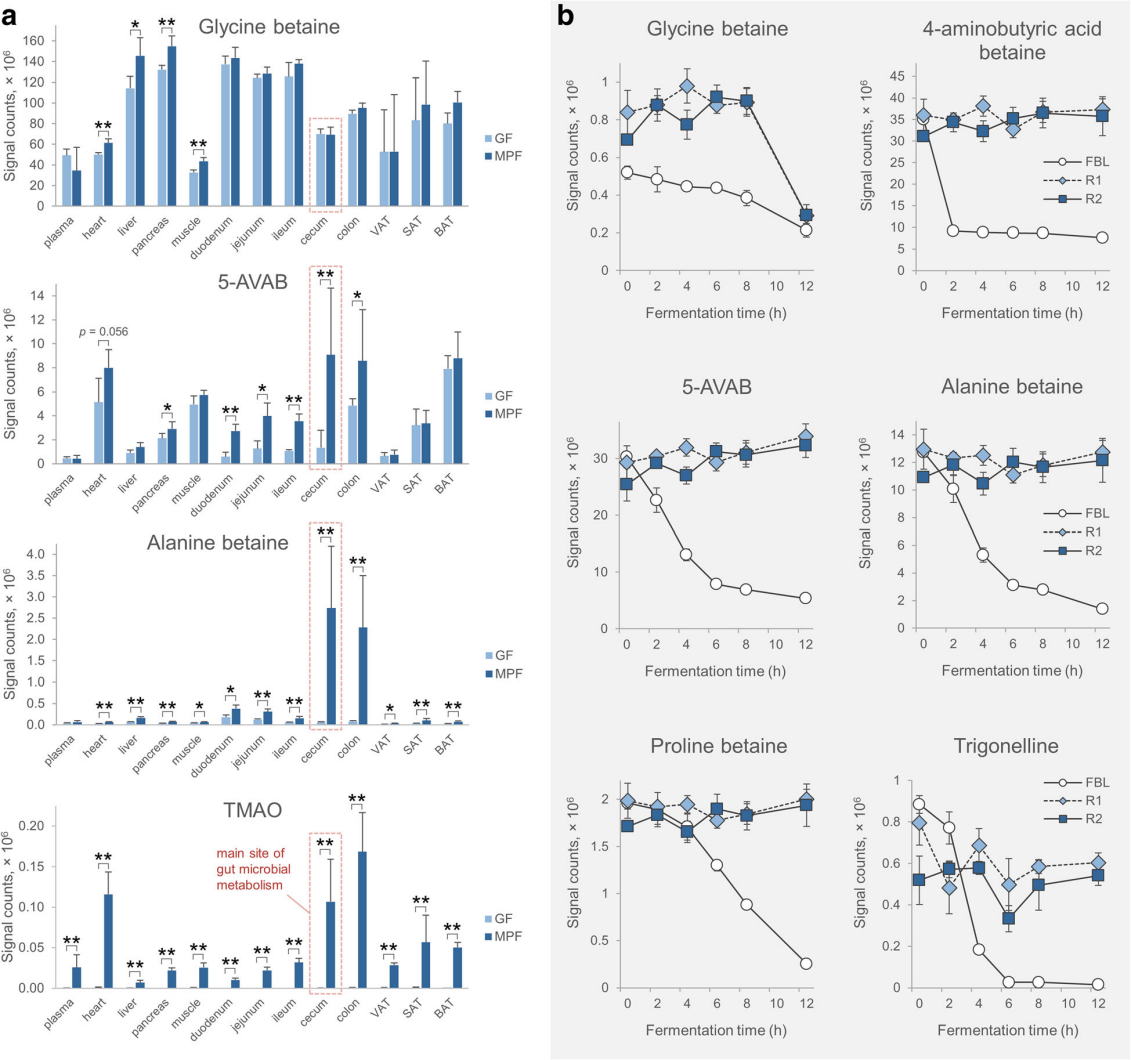
**a** Glycine betaine, 5-AVAB, alanine betaine, and TMAO levels in tissue samples of GF and MPF mice (mean ± 1 SD). Caecal tissue, the main site of gut microbial metabolism in mice, is highlighted. Mann–Whitney *U* test: *p < 0.05; **p < 0.01; ***p < 0.001. **b** Betaine levels in the in vitro 24-h fermentation model with human microbiota, incubated with rye bran fractions (mean ± 1 SD). R1 native rye bran, R2 bioprocessed rye bran, FBL faecal blank

Based on the correlation between betainized compounds and gut microbial taxa, we hypothesized that the colonic microbiota could be the metabolic source for the production of the betainized compounds. We examined 13 different tissue samples from conventional murine pathogen-free (MPF) and germ-free (GF) C57BL/6NTac male mice to support the study hypothesis. The study showed that the levels of glycine betaine were not altered greatly between the mice groups, while the levels of several other betaines, including alanine betaine and 5-AVAB, a betainized metabolite that we characterized recently [37], were significantly lower in the duodenal, jejunal, ileal, and caecal tissues of the germ-free mice compared to conventional mice (Fig. 5a, Additional file 4: Figure S3). In both mice groups, the plasma levels of 5-AVAB were low, whereas its levels in heart, muscle, and BAT were relatively high. Trimethylamine *N*-oxide (TMAO), a betainized metabolite with microbial origin [41], was used as a reference compound to verify the germ-free status of the mice. TMAO was found only as a trace in the tissues of the germ-free mice.

### Microbiota contributes to the production of betainized compounds in a human gut model

After unveiling the potential contribution of microbiota in betaine metabolism, we hypothesized that gut microbiota may transform glycine betaine into other betainized compounds once it reaches the colon by using e.g. fibre matrix as a carrying vehicle. Therefore, we incubated unprocessed and bioprocessed rye bran in an in vitro model of the human gastrointestinal tract with faecal microbiota pooled from volunteer donors to elucidate the time-dependent metabolism of the compounds and to assess whether the observations in mice can be applied to humans. Investigation of samples from the in vitro model of human colonic fermentation showed that similar metabolic conversions can occur in the human gut (Fig. 5b); upon addition of native or bioprocessed rye bran fractions, betainized compounds were produced throughout the 24-h in vitro incubation, whereas the levels of the betainized compounds in the faecal background control samples decreased after 4 to 6 h. In contrast to other betainized compounds, glycine betaine was the only compound to have its level decreased during the 24-h incubation (Fig. 5b).

### Content of betainized compounds in whole-grain breads

Whole grains are a main source of glycine betaine in the Western diet [42]. We have earlier shown that the levels of other betainized compounds were also elevated after the consumption of diets rich in whole grains or cereal bran in humans and mice [35, 36, 43]. Therefore, we examined wheat and rye samples (*n* = 10, prepared at VTT Technical Research Centre of Finland Ltd.) containing processed or whole-grain flour, including rye and wheat flour and breads baked with or without sourdough fermentation. The purpose herein was to find out which betainized compounds other than glycine betaine could be originated from bread. In addition to glycine betaine, relatively low levels of trigonelline, valine betaine, proline betaine, and nine other betaines were found in the bread samples, and their levels were depending on the cereal species and whole grain content (Additional file 5: Figure S4). 5-AVAB, phenylalanine betaine, and tryptophan betaine are noteworthy because they were not detected from the whole-grain bread samples containing the bran fractions (Additional file 1: Table S1), indicating they may be synthesized by microbiota after the ingestion of the bran-enriched diet. Pipecolic acid betaine was nearly absent from all the wheat samples, whereas isoleucine betaine was mainly found in whole-grain wheat bread. Trigonelline was present in higher levels in the flour samples compared to the bread samples, indicating the effect of the baking process. Of the identified betaines, histidine betaine, alanine betaine, isoleucine betaine, and 4-aminobutyric acid betaine have not previously been reported from cereals.

## Discussion

In this study, we observed how the bran-enriched diets elevated the levels of betainized compounds in the colonic content and caecal tissues of mice and altered the gut microbial composition. These results were supported by the low content or lack of betainized compounds (other than glycine betaine) in the studied whole-grain breads, the decreased levels of the same compounds in the gut tissues of germ-free mice compared to conventional mice, and the production of the compounds in an in vitro model of the human gut.

The bran-enriched diets had a pronounced effect on the composition of microbiota in the mice and showed a strong association with an increased abundance of several microbial taxa, including *Akkermansia*, *Bacteroides*, *Bifidobacterium*, an uncharacterized genus within the *Coriobacteriaceae*, *Enterorhabdus*, *Lactobacillus*, *Parasutterella*, and *Ruminococcus*, all of which have previously been associated with an improved metabolic status. Among them, the most well-known bacteria in terms of human health are bifidobacteria and lactobacilli, which are well-recognized as major contributors to improved gut and overall health [44] and have previously been associated with whole-grain wheat intake [25]. *Akkermansia muciniphila*, the sole characterized representative of its phylum in mammalian gut environments, was strongly associated with the bran-enriched diets in our study (Fig. 3) and has previously been shown to be related to decreased fasting glucose and plasma triglycerides in a human intervention study involving a calorie-restricted diet [45]. Similarly, an increase in *Akkermansia* population and metabolic improvements were observed in mice fed with a high-fat diet containing whole-grain barley [46] and cranberry extract [47]. However, the genus is also increased in abundance among multiple sclerosis patients, suggesting that it may have a role in (or increase as a consequence of) the pathogenesis of autoimmune diseases [48]. Members of the family *Coriobacteriaceae* carry out conversions of bile salts, steroids and polyphenols, and are involved in the metabolism of amino acids, nitrogen cycling, and the production of ammonia [49]. This suggests that these bacteria could partly be responsible for the production of amino acid-derived betaines, as they were positively correlated with some of the identified betaines in the current study. *Coriobacteriaceae* are associated with resistance to obesity and liver pathologies during the consumption of a high-fat diet [50]. *Enterorhabdus*, a genus within the *Coriobacteriaceae* and associated with bran-enriched diets in this study, was shown to be specific to microbiota in lean animals [50]. Some genera within *Coriobacteriaceae* may be involved in the development of various pathologies, including infections outside of the GI tract and tumorous growths, indicating that the family should be considered as pathobionts (potentially pathogenic commensal bacteria) [49]. *Ruminococcus* species, such as *R. bromii*, are key microorganisms in the degradation of resistant starch [51], which likely explains their increased relative abundance in the bran-enriched diet groups in this study. Increased abundance of the genus *Parasutterella*, which increased especially after the A4 diet (enriched with bioprocessed wheat aleurone), was previously shown to be inversely associated with a high-fat diet in mice [52]. However, this genus was also found to be associated with certain pathogenic conditions, such as irritable bowel syndrome [53]. *Bacteroides* were increased in their relative abundance after the consumption of bran-enriched diets; they are a predominant bacterial group with health-promoting properties, such as the downregulation of inflammatory biomarkers [54]. Nevertheless, their increased abundance has also been associated with type I diabetes and celiac disease, and some *Bacteroides* species (e.g. *B. ovatus*) have been positively correlated with gastrointestinal dysbiosis and Crohn’s disease [54].

In contrast, the bran-enriched diets reduced the relative abundance of several bacterial taxa that have earlier been associated with adverse effects in the gut and overall health. *Alistipes* was positively associated with frailty in the elderly in a study by Claesson et al. [55]. *Desulfovibrio* species were found to be associated with regressive autism [56] and ulcerative colitis [57] and can be potentially harmful if their abundance in the gut is increased. It has been shown that *Rikenella* and *Odoribacter* were exclusively present in the gut of type 2 diabetic (*db*/*db*) mice when compared to healthy lean mice [58]. In a recent animal study, Song et al. showed that *Mucispirillum, Desulfovibrio*, and *Odoribacter* were strongly correlating with genes overexpressed in colitis-associated cancer [59]. Overall, although several microbial groups have been associated with either beneficial metabolic status or disease, much of the reported results are controversial. We still do not fully understand the impact of individual bacterial species and strains on human health, which is also affected by the whole microbial community and the physiological and nutritional status of the host.

The cereal species seems to have an impact both on the quality and quantity of the gut microbiota, as evidenced by the lower alpha diversity in the caecal microbiota of the C57BL/6 J mice receiving wheat aleurone-enriched diets compared to other diets and the difference in the relative abundance of certain bacterial genera between the rye bran and wheat aleurone-enriched diets. Bran bioprocessing releases high amounts of phenolic compounds from the fibre matrix [40], which may be already absorbed in the small intestine and thus not reach the site of microbial fermentation, where they could be used as a substrate by certain microbes (e.g. *Bifidobacterium*) and thus promote their growth [60]. This may explain why *Lactobacillus* and *Bifidobacterium*, known to ferment insoluble dietary fibre, had lower relative abundance in mice receiving bioprocessed wheat aleurone. Although sourdough fermentation with *Lactobacillus* species was used in some of the cereal samples analyzed for betaine levels, it only had a minor effect in the levels of betainized compounds, including glycine betaine. The low levels or lack of betainized compounds, apart from glycine betaine, in all the studied cereal samples indicates that their metabolism mostly occurs after ingestion.

Glycine betaine is the predominant betainized compound in cereal grains, and thus we expected to observe high levels of the compound in the gut contents of mice and in vitro human colonic model promoted by the bran-enriched diets. However, the addition of bran in the diet did not considerably increase the level of glycine betaine in the colon contents of mice. In contrast, the levels of alanine betaine and valine betaine, which are present in relatively small amounts in bran, and 5-aminovaleric acid betaine (5-AVAB), phenylalanine betaine, and tryptophan betaine, which are completely absent from bran, were increased significantly. Several betainized compounds, such as 5-AVAB and alanine betaine, were also observed at lower levels in the gut tissues (i.e. duodenum, jejunum, ileum, cecum, and colon) of the GF mice compared to MPF mice, indicating that the gut microbiota has an impact on their production, although they may also be acquired from the diet in low amounts. The low level of 5-AVAB in the plasma and its higher levels in the heart, muscle, and BAT indicate that the compound is rapidly transported into these metabolically active tissues after absorption, likely by the cell membrane carnitine transporter to which 5-AVAB is a substrate [37]. Great variability in the levels of 5-AVAB, alanine betaine, and TMAO compared to glycine betaine was observed in the gut tissue samples, which may indicate the microbial origin of these metabolites and could be explained by the variation in the gut microbial composition (Additional file 6: Figure S5). In the in vitro incubation containing microbiota from human donors, a similar trend was evident, as the level of glycine betaine decreased while the level of several other betainized compounds, including 5-AVAB, alanine betaine, and proline betaine, remained constant throughout the incubation in the rye bran-containing samples while their levels decreased in the control samples. These findings suggest that components in rye bran may supply the production of betainized compounds and that glycine betaine is likely the main source of the metabolites. Furthermore, the results strongly suggest that microbiota contributes to the production of these compounds, supported by the location of the metabolism in the gut, the correlation between the abundance of betainized compounds and the bacterial taxa that were increased by the bran-enriched diet, and the lowered levels of these compounds in the tissues of the germ-free mice.

## Conclusions

We showed that a group of betainized compounds, which were increased in abundance by dietary cereal bran, were associated with the composition of gut microbiota. According to our findings, bran-enriched diet can increase the relative abundance of beneficial gut microbes while decreasing the abundance of bacteria associated with adverse health effects. At the same time, the bran acted as a source of glycine betaine, which was later metabolized into other betainized compounds, a process likely contributed by the same gut microbes that were favourably affected by the bran-enriched diet. The importance of gut microbiota as a mediator of the metabolic effect of diet quality is increasingly highlighted, and the study presented here brings out yet another metabolically relevant class of compounds modulated by the diet and microbiota. Further mechanistic studies with stable isotope labelled glycine betaine incubated with selected bacteria are needed to verify the metabolic pathways of betaines in gut microbiota. In addition, the potential and mechanism(s) by which betainized compounds maintain the homeostasis and improve health merit continued investigation.

## Methods

### Animal experiments

The animal experiments were approved by the Institutional Animal Care and Use Committee of the Provincial Government of Finland (license number 041003). C57BL/6 J male mice (*n* = 74) were obtained from the National Laboratory Animal Center (Kuopio, Finland) at the age of 9 weeks and kept under conditions as described by Pekkinen et al. [36]. After 1 week of acclimatization, the mice (*n* = 64) were fed ad libitum a commercial high-fat diet (D12451, Research Diets Inc., USA) for 9 weeks to induce obesity. Furthermore, one control group (*n* = 10) was fed ad libitum a commercial low-fat control diet (D12450B, Research Diets Inc., USA). After 9 weeks of pre-feeding, the mice (except for the LF group) were randomized into three control and four study groups (*n* = 9–14) and fed either with D12451, D12450B, or HF diets containing the bran enrichment. The rye bran-enriched diets contained either unprocessed rye bran (R1) or bioprocessed, i.e. enzymatically treated and yeast fermented rye bran (R2), whereas the wheat aleurone-enriched diets contained untreated wheat aleurone (A1) or ground and enzymatically treated (xylanase and ferulate esterase) aleurone (A4). A more detailed description of the diets has been published previously [36, 61]. The breads were tailor-made at VTT Technical Research Centre of Finland Ltd, as described previously [62]. Table 1 shows an overview of the dietary intervention. After the feeding trial, the mice were fasted for 8 ± 0.5 h and sacrificed by decapitation after being made unconscious by CO_2_ gas. The tissues and intestinal tissue contents were collected. The tissue contents were separated from tissues, stored in microcentrifuge tubes, and frozen on dry ice. The tissues were rinsed with physiological saline, wrapped in aluminium foil, and snap frozen in liquid nitrogen. All samples were kept at − 80 °C until further processing.

**Table 1:** Study groups and the diets of the C57BL/6 J mice in the bran-enriched feed trial

Study group	*n*	Week 1–9	Week 10–18
HF	14	HF (D12451)	HF (D12451)
HFLF	9	HF (D12451)	LF (D12450B)
LF	10	LF (D12450B)	LF (D12450B)
R1	11	HF (D12451)	D12451 + unprocessed rye bran
R2	10	HF (D12451)	D12451 + bioprocessed rye bran
A1	9	HF (D12451)	D12451 + unprocessed wheat aleurone
A4	11	HF (D12451)	D12451 + bioprocessed wheat aleurone

Conventional murine pathogen free (*n* = 5) and germ-free (*n* = 5) C57BL/6NTac male mice aged 10 weeks were obtained from Taconic Biosciences, Inc. (Hudson, USA). They were kept on a sterile NIH-31 rodent diet. Blood was collected and centrifuged at 3000*g* for 10 min at room temperature. The 12 other tissue samples (heart, liver, pancreas, muscle, duodenum, jejunum, ileum, cecum, colon, VAT, SAT, and BAT) were rinsed with physiological saline. All tissues were snap-frozen and kept in − 80 °C until processing for metabolomics analyses.

#### Sample processing

Frozen tissue samples were cryo-ground either in 10 ml grinding steel jars containing a stainless steel ball for 60 s with 15 Hz (liver, subcutaneous adipose tissue) or in 2 ml microcentrifuge tubes containing a 4 or 7 mm stainless steel bead in a pre-cooled 2 × 24 adapter that was shaken for 45 s at 30 Hz (BAT, muscle, heart) using TissueLyser II (Qiagen Finland, Helsinki, Finland). Samples containing 100 mg (± 2 mg) of tissue powder were cryo-weighted into 1.5 mL microcentrifuge tubes and 90% methanol was added (v/v H_2_O, LC-MS Ultra CHROMASOLV®, Fluka) in a ratio of 300 μL solvent per 100 mg tissue. The samples were shaken for 20 min. Colon contents were weighed, mixed with 90% methanol (500 μl methanol per 100 mg content), and shaken with 4-mm stainless steel beads for 45 s at 20 Hz. All samples were centrifuged for 10 min at 4 °C (13 000 rpm), and supernatants were filtered using 0.2 μm Acrodisc® Syringe Filters with a PTFE membrane (PALL Corporation) and stored at − 20 °C until the LC–MS analysis. The bread samples were processed as described previously [62].

#### Cereal samples and in vitro colonic fermentation

The processed and whole-grain cereal samples were prepared according to Koistinen et al. [62]. The starter used for the sourdough fermentation contained baker’s yeast (*Candida milleri*) 10^6^ cfu/g for whole-grain wheat and 10^7^ cfu/g for whole-grain rye and lactic acid bacteria (*Lactobacillus brevis* and *Lactobacillus plantarum*) 10^7^ cfu/g for whole-grain wheat and 10^8^ cfu/g for whole-grain rye for both bacterial species. The in vitro upper intestinal model is described in detail by Aura et al. [63] and the colonic model by Nordlund et al. [64]. Briefly, to mimic the digestion in the upper intestinal tract, wheat breads fortified with native (unprocessed) or bioprocessed rye bran were first ground in a mincer (MG450, Kenwood Ltd, Hampshire, UK) and then exposed to enzymatic digestion using porcine enzymes (salivary α-amylase, pepsin, and pancreatin). The digestion products were removed by dialysis, after which the non-digested residues were freeze-dried and moved into an in vitro colonic model. A faecal suspension (10% *w*/*v*) collected and pooled from 5 healthy volunteers was used for the incubation, performed in strictly anoxic conditions. Samples were taken during the fermentation at time points 0, 2, 4, 6, 8, and 24 h.

#### LC–MS experiments

The mouse tissue, colon contents, cereal, and in vitro fermented samples were analyzed by ultra-high-performance liquid chromatography quadrupole time-of-flight mass spectrometry (UHPLC–qTOF-MS) system (Agilent Technologies), consisting of a 1290 LC system, a Jetstream electrospray ionization (ESI) source, and a 6540 UHD accurate-mass qTOF spectrometer. The chromatographic separation was performed with hydrophilic interaction chromatography (HILIC). The sample tray was kept at + 4 °C during the analysis. The data acquisition software was the MassHunter Acquisition B.04.00 (Agilent Technologies). During the liquid chromatography, 3 μL of the sample solution was injected onto the column (Acquity UPLC BEH Amide column, 2.1 × 100 mm, 1.7 μm; Waters Corporation) and maintained at 45 °C. The mobile phases, delivered at 0.6 mL/min, consisted of 50% acetonitrile (vol:vol; eluent A) and 90% acetonitrile (vol:vol; eluent B), respectively, both containing 20 mmol/L ammonium formate, pH 3 (Sigma-Aldrich). The following gradient profile was used: 0–2.5 min, 100% B; 2.5–10 min, 100% B → 0% B; 10–10.1 min, 0% B → 100% B; 10.1–14 min, 100% B.

The MS conditions were as follows: Jetstream ESI source, operated in positive ionization mode, with a drying gas temperature of 325 °C and flow of 10 L/min, a sheath gas temperature of 350 °C and flow of 11 L/min, a nebulizer pressure of 310 kPa, capillary voltage of 3500 V, nozzle voltage of 1000 V, fragmentor voltage of 100 V, and a skimmer voltage of 45 V. For data acquisition, a 2 GHz extended dynamic range mode was used, and the instrument was set to acquire ions over the *m*/*z* range 50–1600. Data were collected in the centroid mode at the acquisition rate of 2.5 spectra/s (i.e. 400 ms/spectrum) with an abundance threshold of 150 counts. QC samples were used for the automatic data-dependent MS/MS analyses. From every precursor scan cycle, 4 most abundant ions were selected for fragmentation. These ions were excluded after 2 product ion spectra and released again for fragmentation after a 0.25-min hold. The precursor scan time was based on ion intensity, ending at 20,000 counts or after 300 ms. The product ion scan time was 300 ms. The collision energies were 10, 20, and 40 V in subsequent assays. The continuous mass axis calibration was performed by monitoring two reference ions (*m*/*z* 121.050873 and *m*/*z* 922.009798) from an infusion solution throughout the assays.

#### Microbial DNA extraction, 16S ribosomal RNA gene sequencing, and microbial composition analysis

Total bacterial DNA was extracted using the Maxwell® 16 Total RNA system (Promega) with Stool Transport and Recovery Buffer (STAR; Roche Diagnostics Corporation, Indianapolis, IN). Briefly, 0.15 g of caecal content sample was homogenized with 0.25 g of sterilized 0.1 mm zirconia beads and three glass beads (2.5 mm) in 350 μL STAR buffer for 3 min at 5.5 ms. Samples were incubated with shaking at 100 rpm for 15 min at 95 °C and pelleted by 5 min centrifugation at 4 °C and 14 000 g. The supernatant was removed, and the pellets were processed again using 200 μL of fresh STAR buffer. The supernatant was again removed and pooled and 250 μL was used for purification with Maxwell® 16 Tissue LEV Total RNA Purification Kit (AS1220) following manufacturer’s instructions. DNA was eluted with 50 μL of DNAse and RNAse free water (Qiagen, Hilden, Germany). DNA concentrations were measured with NanoDrop ND-1000 (NanoDrop® Technologies, Wilmington, DE, USA) and adjusted to 20 ng/μL with DNAse and RNAse free water. The V4 region of 16S ribosomal RNA (rRNA) genes was amplified. PCR reactions were done in duplicates, each in a total volume of 50 μL and containing 20 ng of template DNA. Each sample was amplified with a unique barcoded primer 515F-n and 806R-n (10 μM each/reaction), 1× HF buffer (Finnzymes, Vantaa, Finland), 1 μL dNTP Mix (10 mM each, Roche Diagnostics GmbH, Mannheim, Germany), 1 U Phusion® Hot Start II High Fidelity DNA Polymerase (Finnzymes, Vantaa, Finland), and 36.5 μL of DNAse and RNAse free water. The amplification programme included 30 s initial denaturation step at 98 °C, following by 25 cycles of denaturation at 98 °C for 10 s, annealing at 56 °C for 10 s, elongation at 72 °C for 10 s, and a final extension at 72 °C for 7 min. The PCR product presence and size (~ 290 bp) was confirmed with gel electrophoresis using the Lonza FlashGel® System (Lonza, Cologne, Germany). Unique barcode tags (*n* = 70) were used in each library and artificial control (Mock) communities were included, as described previously [65]. PCR products were purified with HighPrep® PCR kit (MagBio Genomics, Alphen aan den Rijn, Netherlands), and DNA concentrations were measured with Qubit® dsDNA BR Assay Kit (Life Technologies, Leusden, Netherlands). One hundred nanograms of each barcoded sample was pooled together, the amplicon pool was concentrated with HighPrep® PCR kit to 20 μL volume, and the concentration was measured with Qubit® dsDNA BR Assay Kit and adjusted to 100 ng/μL final concentration. The libraries were sent for adapter ligation and HiSeq sequencing (GATC-Biotech, Konstanz, Germany).

#### Data analysis

For the betaine metabolites, data processing was done using Profinder (Agilent Technologies), which enabled manually picking up peaks associated with the amino acid-derived betaines from the mass spectrometry data. Betaines were identified by comparison of retention times and use of targeted MS/MS spectra, which were compared to spectra of commercial and synthetized chemical standards (4-aminobutyric acid betaine, 5-AVAB, alanine betaine, glycine betaine, phenylalanine betaine, pipecolic acid betaine, proline betaine, trigonelline, tryptophan betaine, valine betaine) or published spectra (other betaines).

For the statistical analysis, we used parametric one-way ANOVA for comparison between all the diet groups for the betaine levels, Mann–Whitney *U* test for comparison between germ-free and conventional mice for the organ-specific betaine levels and for pairwise comparisons of the relative bacterial abundances between the diet groups, and Kruskal–Wallis one-way ANOVA for comparison between relative bacterial abundances between all diet groups. The α level was set at 0.05 for all statistical tests. For the microbial composition analysis, data processing and analysis was carried out using in-house developed scripts [65]. The samples were rarefied at 3900 reads prior to the alpha diversity analyses (Shannon’s index and Simpson diversity index), which were carried out in QIIME [66]. To determine the difference between the impact of control and treatment diets on the relative microbial abundance, we performed ANOSIM analyses in QIIME using weighted and unweighted UniFrac distances. The redundancy analyses (RDA) were performed in Canoco5 [67]. In RDA, the variance between the samples (genus-level relative bacterial abundance) is explained by environmental variables (in this case diet and betainized metabolites), which were fitted to corresponding matrices in the resulting illustration. PCA was performed in RStudio v. 1.1.447 using in-house scripts. The Pearson correlation between bacterial taxa and betaines, Kruskal–Wallis one-way ANOVA, Mann–Whitney *U* test, and the fold change analyses between the diet groups were performed in SPSS 25 (IBM) and plotted with an in-house R script utilizing ggplot2 package. The microbial abundance heatmap was produced in Multiple Experiment Viewer v4.9.0. For this purpose, the relative abundances were first normalized based on the following formula: *x* = (*x* − $\overline{x}$_row_)/SD_row_.

## Additional files

Additional file 1: **Table S1.** Betainized compounds observed in the current study with their LC–MS 487 identification characteristics and presence in each studied sample type. In case the compound was 488 detected only as trace in a sample, the identification was based on exact m/z and retention time. 1 = 489 identified with a reference standard; 2 = putatively annotated based on publicly available exact m/z 490 and MS/MS spectra. (DOCX 154 kb)

Additional file 2: **Figure S1.** Principal component analysis (PCA) and redundancy analysis (RDA) of the microbial composition (relative abundance data) from the caecal contents of the C57BL/6J mice, with the control diet groups (left) and treatment groups (right) treated separately. The RDA illustrations display 10 best-fitting microbial genera. (TIF 906 kb)

Additional file 3: **Figure S2.** Box plots of selected bacterial genera in the caecal contents of the C57BL/6J mice with significantly different relative abundances in Kruskal–Wallis one-way ANOVA between all diet groups. Outliers are marked with a circle (○) and extreme outliers with an asterisk. (TIF 411 kb)

Additional file 4: **Figure S3.** The average abundance (as signal counts) of all the identified betainized compounds in the tissue samples of GF and MPF mice. The error bars signify an error of 1 SD. Asterisks based on Mann–Whitney U test between the groups: *p < 0.05; ** *p* < 0.01; ***p < 0.001. (TIF 1126 kb)

Additional file 5: **Figure S4.** The levels (as signal counts) of betainized compounds detected with UHPLC–qTOF-MS in 10 different wheat and rye samples, including processed (white or sifted, containing only the endosperm) and whole-grain flour (containing all the edible parts of the grains in their original proportions), processed and whole-grain breads, and sourdough fermented breads tailor-made at VTT Technical Research Centre of Finland. The error bars signify an error of 1 SD. (TIF 592 kb)

Additional file 6: **Figure S5.** The relative abundance of bacterial phyla in the caecal contents of the studied C57BL/6J mice, divided into control diet groups and those fed with the bran-enriched diets. The samples are arranged in ascending order of the most abundant phylum, Firmicutes, which ranges from 52% to 93% of the microbial population. (PNG 113 kb)

## Data Availability

The datasets analyzed in the current study are available from the B2SHARE data repository maintained by the European research data infrastructure and service unit (EUDAT); DOI: 10.23728/b2share.648f1de094d84c40a3b17016b8a870c8. The scripts used in the processing of 16S rRNA sequencing data are available at http://github.com/JavierRamiroGarcia/NG-Tax.
