# Supplementary figures and images for: Contribution of gut microbiota to metabolism of dietary glycine betaine in mice and in vitro colonic fermentation
Source: Microbiome. 2019 Jul 10;7:103. doi: 10.1186/s40168-019-0718-2 (PMC6621954; doi:10.1186/s40168-019-0718-2)

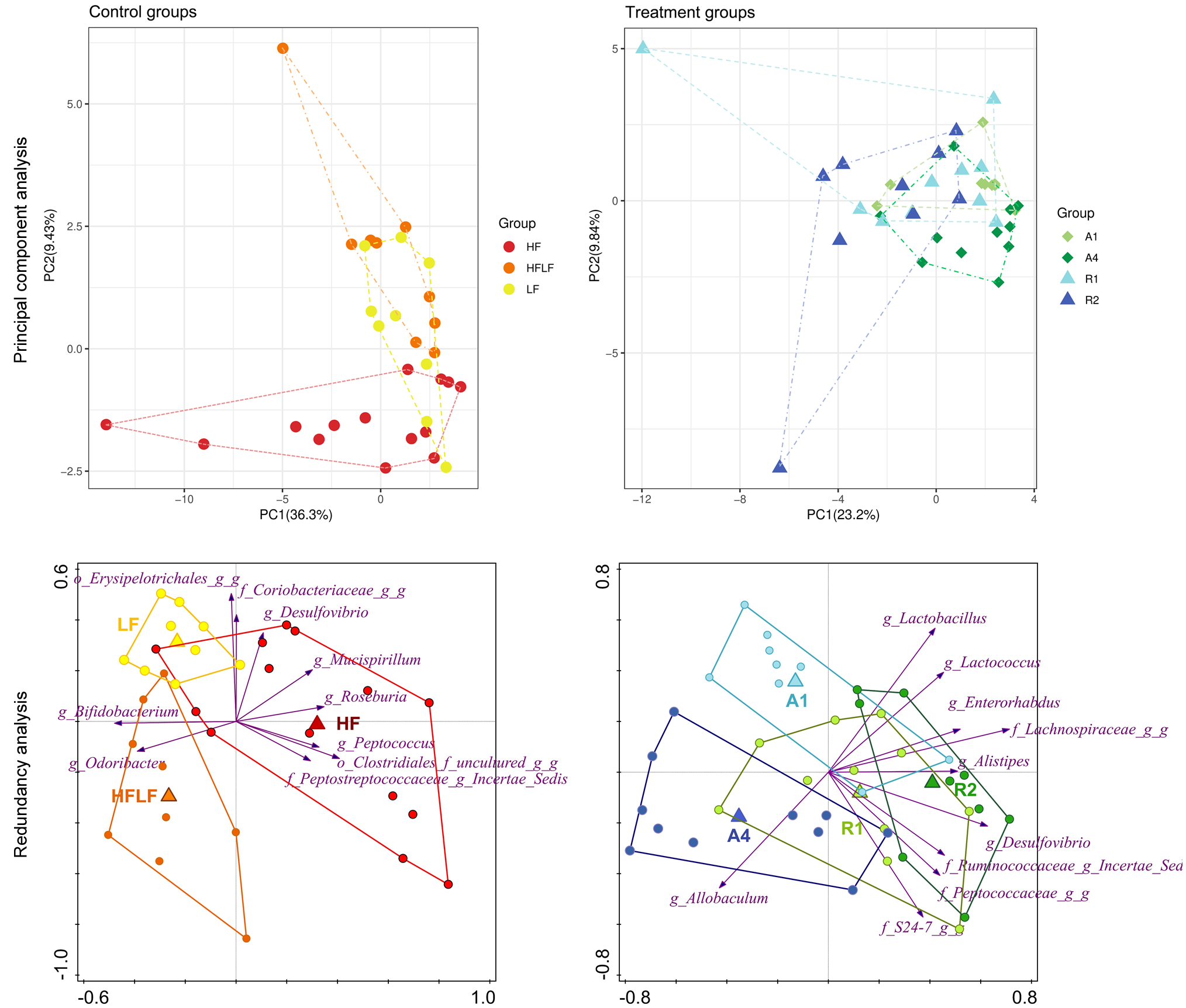

Supplement: Supplementary file 2 — Figure S1. Principal component analysis (PCA) and redundancy analysis (RDA) of the microbial composition (relative abundance data) from the caecal contents of the C57BL/6J mice, with the control diet groups (left) and treatment groups (right) treated separately. The RDA illustrations display 10 best-fitting microbial genera. (TIF 906 kb) [file 40168_2019_718_MOESM2_ESM.tif]

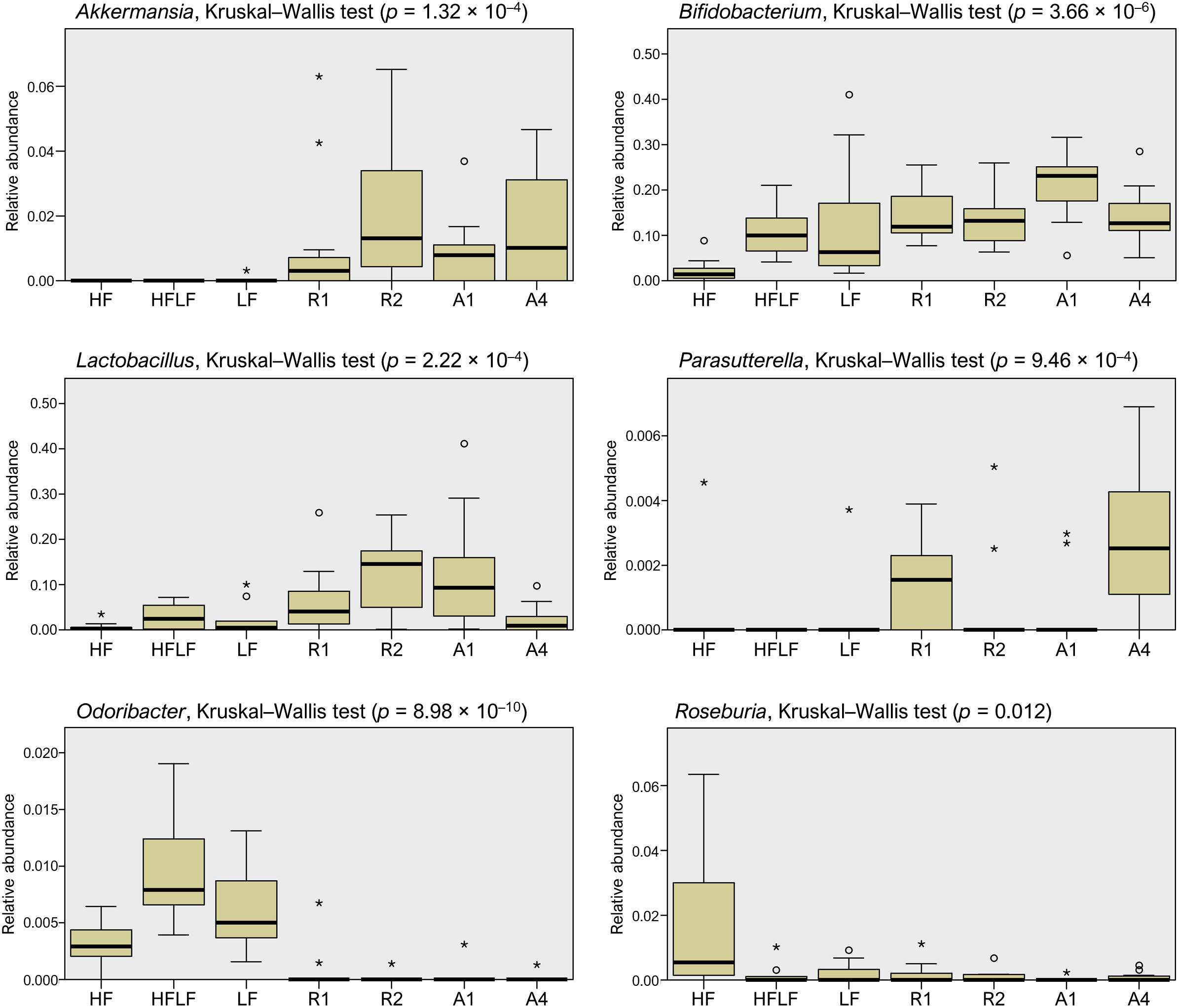

Supplement: Supplementary file 3 — Figure S2. Box plots of selected bacterial genera in the caecal contents of the C57BL/6J mice with significantly different relative abundances in Kruskal–Wallis one-way ANOVA between all diet groups. Outliers are marked with a circle (○) and extreme outliers with an asterisk. (TIF 411 kb) [file 40168_2019_718_MOESM3_ESM.tif]

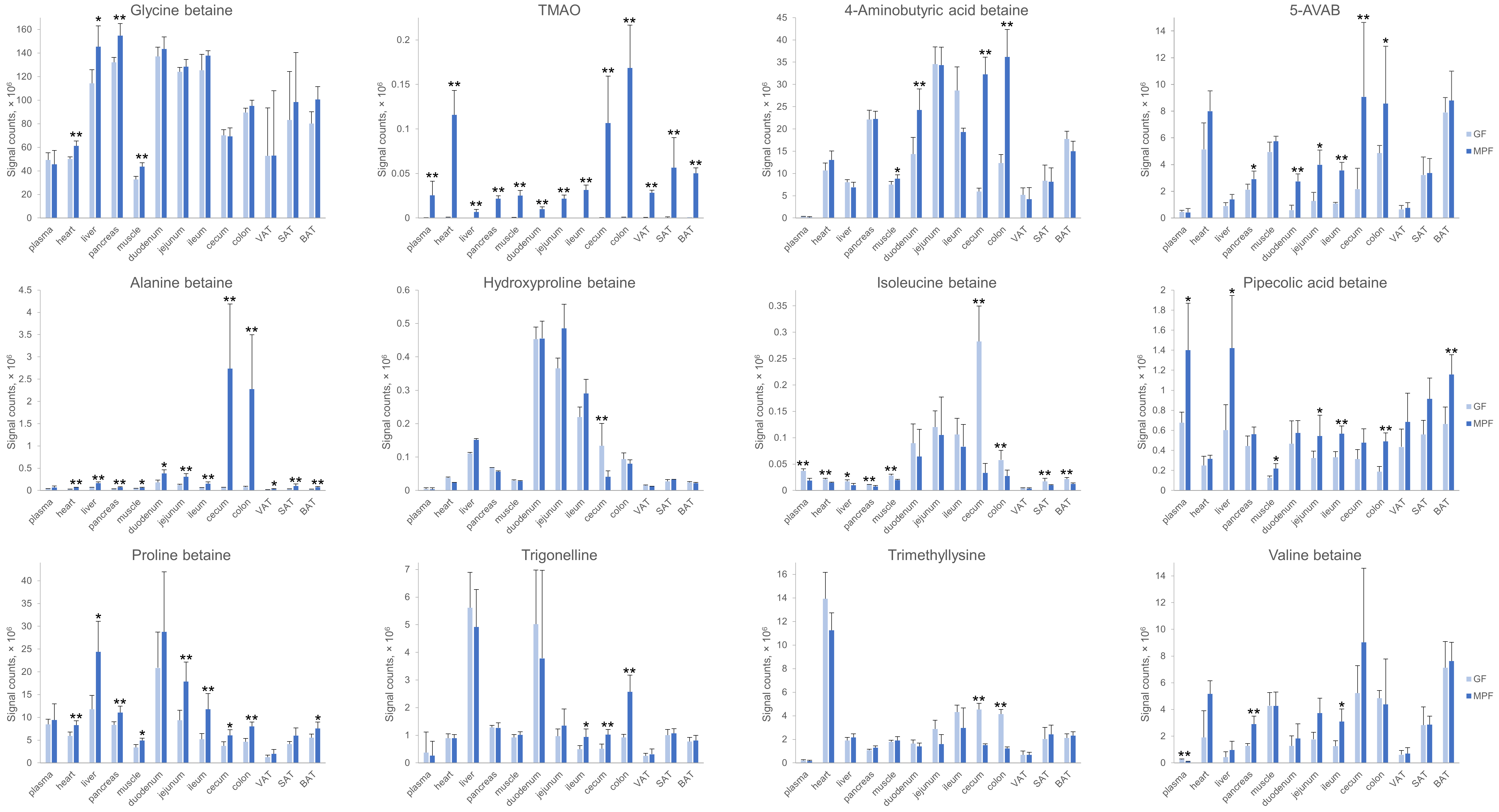

Supplement: Supplementary file 4 — Figure S3. The average abundance (as signal counts) of all the identified betainized compounds in the tissue samples of GF and MPF mice. The error bars signify an error of 1 SD. Asterisks based on Mann–Whitney U test between the groups: *p < 0.05; ** p < 0.01; ***p < 0.001. (TIF 1126 kb) [file 40168_2019_718_MOESM4_ESM.tif]

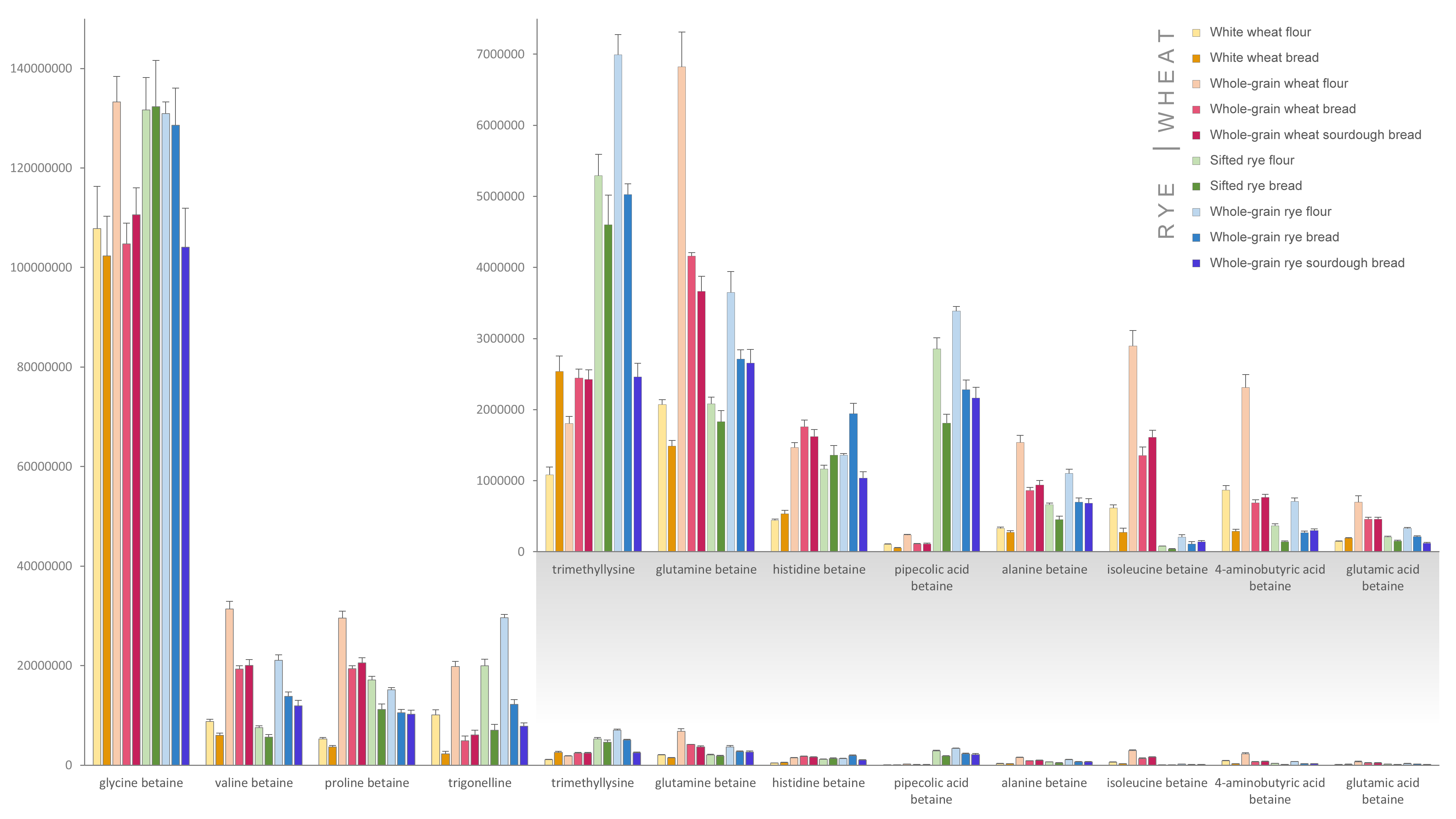

Supplement: Supplementary file 5 — Figure S4. The levels (as signal counts) of betainized compounds detected with UHPLC–qTOF-MS in 10 different wheat and rye samples, including processed (white or sifted, containing only the endosperm) and whole-grain flour (containing all the edible parts of the grains in their original proportions), processed and whole-grain breads, and sourdough fermented breads tailor-made at VTT Technical Research Centre of Finland. The error bars signify an error of 1 SD. (TIF 592 kb) [file 40168_2019_718_MOESM5_ESM.tif]

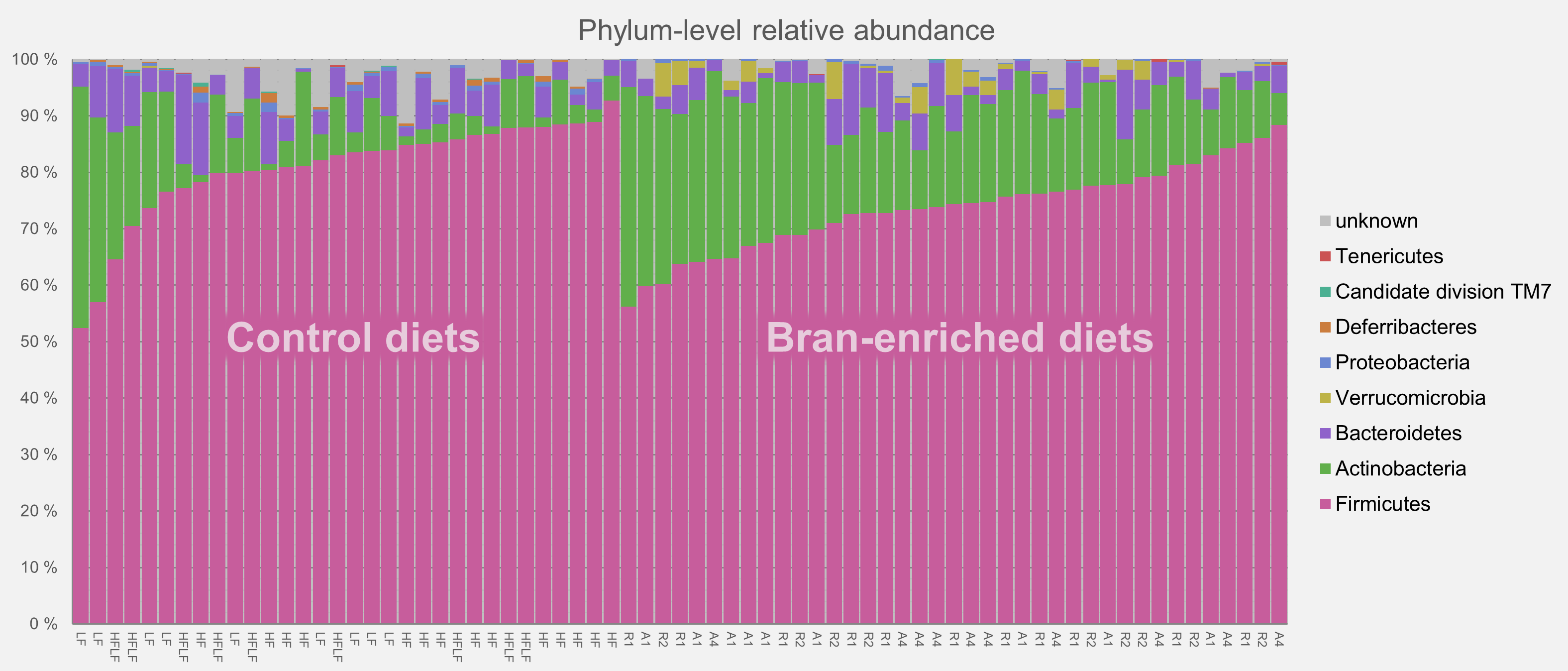

Supplement: Supplementary file 6 — Figure S5. The relative abundance of bacterial phyla in the caecal contents of the studied C57BL/6J mice, divided into control diet groups and those fed with the bran-enriched diets. The samples are arranged in ascending order of the most abundant phylum, Firmicutes, which ranges from 52% to 93% of the microbial population. (PNG 113 kb) [file 40168_2019_718_MOESM6_ESM.png]
