# Supplementary material for: Contribution of gut microbiota to metabolism of dietary glycine betaine in mice and in vitro colonic fermentation
Source: Microbiome. 2019 Jul 10;7:103. doi: 10.1186/s40168-019-0718-2 (PMC6621954; doi:10.1186/s40168-019-0718-2)
Supplement: Supplementary file 2 [file 40168_2019_718_MOESM1_ESM.docx]

Supplementary Table 1. Betainized compounds observed in the current study with their LC–MS identification characteristics and presence in each studied sample type. In case the compound was detected only as trace in a sample, the identification was based on exact *m/z* and retention time. 1 = identified with a reference standard; 2 = putatively annotated based on publicly available exact *m/z* and MS/MS spectra.

	Observed characteristics in LC–MS (HILIC+)				Identification in samples				
**Compound**	***m/z***	**Δppm**	**RT [min]**	**MS/MS spectrum [CID voltage: *m/z* (intensity%)]**	**Colon contents**	**MPF mice**	**GF mice**	**Human *in vitro***	**WG bread**
TMAO	76.0758	−5.8	1.48	20 V: 58.065 (100), 59.073 (60), 76.076 (21; [M + H]$^+$)	n/d	1	trace	n/d	n/d
Glycine betaine	118.0863	−4.2	3.39	20 V: 58.066 (100), 59.074 (76), 118.087 (26; [M + H]$^+$)	1	1	1	1	1
Alanine betaine	132.1019	−8.3	3.21	20 V: 59.073 (100), 58.065 (81), 81.054 (7)	1	1	1	1	1
Trigonelline	138.0552	−6.2	3.94	20 V: 138.055 (100; [M + H]$^+$), 92.049 (91), 94.065 (83), 53.039 (20), 78.034 (19), 65.039 (15)	1	1	1	1	1
Proline betaine	144.1019	−7.6	3.28	20 V: 144.103 (100; [M + H]$^+$), 58.066 (44), 84.082 (38)	1	1	1	1	1
4-aminobutyric acid betaine	146.1173	−9.3	3.06	20 V: 43.019 (100), 87.045 (76), 45.034 (42), 60.081 (23), 58.066 (20)	1	1	1	1	1
Pipecolic acid betaine	158.1177	−6.0	2.86	40 V: 58.065 (100), 158.118 (33; [M + H]$^+$), 70.065 (29), 98.095 (26), 56.049 (19), 44.049 (14), 43.041 (14), 42.034 (10)	1	1	1	n/d	1
Hydroxyproline betaine	160.0968	−7.0	4.46	40 V: 58.064 (100), 70.065 (35), 42.034 (24), 82.065 (22)	n/d	1	1	n/d	n/d
Valine betaine	160.1332	−6.9	1.86	20 V: 60.082 (100), 59.074 (45), 58.066 (17), 73.065 (8)	1	1	1	1	1
5-AVAB	160.1334	−5.6	1.94	20 V: 55.055 (100), 60.081 (929, 101.059 (49), 83.049 (33), 43.018 (22), 59.050 (19), 160.133 (12; [M + H]$^+$), 58.064 (6)	1	1	1	1	n/d
Isoleucine betaine	174.1485	−8.3	1.33	20 V: 60.082 (100), 59.074 (45), 58.066 (17), 73.065 (8)	trace	trace	trace	n/d	2
Glutamine betaine	189.1234	−5.6	5.10	20 V: 130.050 (100), 60.081 (84), 85.029 (29), 84.045 (22), 102.056 (17), 41.039 (13), 44.013 (10)	2	n/d	n/d	n/d	2
Trimethyllysine	189.1600	−4.5	6.76	20 V: 84.082 (100), 130.087 (15), 60.081 (13), 144.139 (4)	2	2	2	trace	2
Glutamic acid betaine	190.1073	−6.2	5.55	20 V: 59.073 (100), 60.082 (88), 58.065 (61), 190.107 (30; [M + H]$^+$), 131.034 (9), 113.022 (5)	n/d	trace	trace	trace	2
Histidine betaine	198.1237	−5.6	5.98	20 V: 95.060 (100), 60.081 (34), 68.050 (7), 154.133 (4)	2	2	2	trace	2
Phenylalanine betaine	208.1332	−5.3	1.29	20 V: 107.048 (100), 131.048 (80), 103.054 (62), 58.066 (50), 208.133 (48; [M + H]$^+$), 79.053 (48)	1	trace	trace	1	n/d
Tryptophan betaine	247.1467	6.1	1.32	20 V: 146.061 (100), 188.072 (54), 60.082 (48)	1	n/d	n/d	trace	n/d
